# Evaluation of dimensional accuracy of dental bridges manufactured with conventional casting technique and CAD/CAM system with Ceramill Sintron blocks using CMM

**DOI:** 10.15171/joddd.2018.041

**Published:** 2018-12-19

**Authors:** Alireza Izadi, Fariborz Vafaee, Arash Shishehian, Ghodratollah Roshanaei, Behzad Fathi Afkari

**Affiliations:** ^1^Department of Prosthodontic Dentistry, Faculty of Dentistry, Hamadan University of Medical Sciences, Hamadan, Iran; ^2^Implant Research Center, Department of Prosthodontics, Faculty of Dentistry, Hamadan University of Medical Sciences, Hamadan, Iran; ^3^Modeling of Noncommunicable Diseases Research Center, Department of Biostatistics and Epidemiology, Faculty of Public Health, Hamadan University

**Keywords:** CAD/CAM, Ceramill Sintron, discrepancies, fabrication techniques, fit of prostheses, frameworks

## Abstract

***Background.*** Recently, non-presintered chromium-cobalt (Cr-Co) blocks with the commercial name of Ceramill Sintron were introduced to the market. However, comprehensive studies on the dimensional accuracy and fit of multi-unit frameworks made of these blocks using the coordinate measuring machine (CMM) are lacking. This study aimed to assess and compare the dimensional changes and fit of conventional casting and milled frameworks using Ceramill Sintron.

***Methods.*** A metal model was designed and scanned and 5-unit frameworks were fabricated using two techniques: (I) the conventional casting method (n=20): the wax model was designed, milled in the CAD/CAM machine, flasked and invested; (II) the milling method using Ceramill Sintron blocks (n=20): the wax patterns of group 1 were used; Ceramill Sintron blocks were milled and sintered. Measurements were made on the original reference model and the fabricated frameworks using the CMM in all the three spatial dimensions, and dimensional changes were recorded in a checklist. Data were analyzed with descriptive statistics, and the two groups were compared using one-way ANOVA and Tukey test (α=0.05).

***Results.*** The fabricated frameworks in both groups showed significant dimensional changes in all the three dimensions. Comparison of dimensional changes between the two groups revealed no significant differences (P>0.05) except for transverse changes (arch) that were significantly greater in Ceramill Sintron frameworks (P<0.05).

***Conclusion.*** The two manufacturing processes were the same regarding dimensional changes and the magnitude of marginal gaps and both processes resulted in significant dimensional changes in frameworks. Ceramill Sintron frameworks showed significantly greater transverse changes than the conventional frameworks.

## Introduction

Base-metal alloys currently used for the fabrication of dental prostheses via the conventional manufacturing process are divided into two groups of nickel-chromium (Ni-Cr) and chromium-cobalt (Cr-Co) alloys.^1^ The Ni-Cr alloys can be allergenic due to the release of Ni ions.^2,3^ Thus, Cr-Co alloys are more commonly used for the fabrication of restorations.^4,5^

Recent advances in technology revolutionized the designing and fabrication of dental restorations by the computer-aided design/computer-aided manufacturing (CAD/CAM) systems.^6-8^ Several alloys and materials are used for the fabrication of restorations using the CAD/CAM systems and their quality and variability continuously improve with advances in science and technology.^9^

Due to the hardness of Cr-Co-based alloys, the milling process of fully-sintered blocks is difficult and results in excessive wear of the milling machine. For this reason, presintered soft Cr-Co blocks were recently introduced to enhance the process of fabrication and milling of these restorations.^4^ In the process of fabrication of these restorations, the restoration is first designed and then the respective blocks are milled by the CAD/CAM machine. The restoration then undergoes sintering to attain its final density and hardness.^10^ Acceptable clinical results with the use of fixed dental prostheses partly depends on the proper fit of restoration over the abutment teeth, which directly affects the long-term prognosis of treatment.^11-16^ Restoration misfit and the resultant marginal gap bring about several complications, including cement washout and subsequent periodontal problems, secondary caries and pulpitis.^14-20^

Several studies have evaluated the distortion of restorations and several methods have been proposed and employed to quantify the magnitude of distortion.^21-23^ Evaluation of cement thickness and photoelasticity, simulation with silicon, profilometry and micro-computed tomography are among the methods applied to quantify distortion.^9-10,17,24-26^ Recently, the coordinate measuring machine (CMM) was designed for this purpose, which is one of the most accurate tools for this task.^27,28^ It has 10-µm measurement accuracy and can be used as the gold standard for dimensional measurements.^29^

Previous studies on the dimensional accuracy of restorations fabricated of presintered soft Cr-Co (Ceramill Sintron) blocks have mainly assessed single-unit restorations and have not used the CMM for assessment of the fit of these restorations. Thus, this study aimed to precisely assess the fit of frameworks fabricated with the use of conventional casting method and milled frameworks using the Ceramill Sintron blocks.

The null hypothesis stated that the frameworks fabricated using the two methods would have no difference with each other or the original reference model in terms of dimensional accuracy.

## Methods

**Figure 1:**
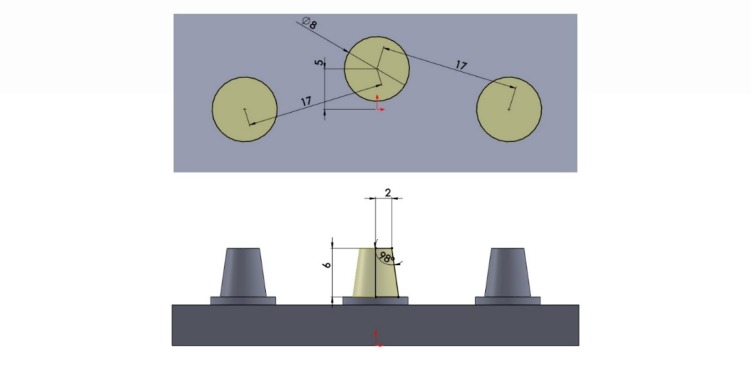
Designing the model.

**Figure 2:**
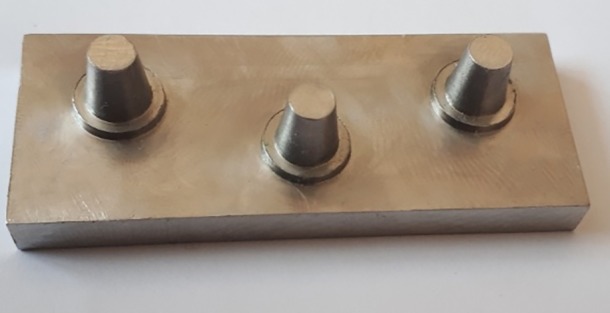
Milled reference model.

A model (representing the part of jaw under treatment) with three distanced prepared abutments that would be eventually restored with a 5-unit bridge (three retainers and two pontics) was designed for this study using SolidWorks 2016 software program as follows. Each abutment was 6 mm in height and 4 mm in diameter at the occlusal area, with a 1-mm shoulder and 8° taper. The first and third abutments were positioned in a horizontal plane and the second abutment was positioned 5 mm external to this plane in y axis so that the center of the occlusal surface of the second abutment was 12 mm away from the center of the occlusal surface of abutments #1 and #3 (Figures 1 and Figure 2).

This design was transferred to a CNC machine (Maschinen, Wagner, Germany) and the model was milled of 316 steel alloy (UNS S31600, AK Steel, USA).

The sample size was calculated to be 20 according to a study by Hjalmarsson et al,^30^ assuming σ1=15, σ2=8, d=11.5, type one error of 5% and a power of 80% using the following sample size calculation formula (1):

[1] $$n=\frac{\left(Z_{1-\frac{\alpha }{2}}+Z_{1-\beta })(\sigma_{1}^{2}+\sigma_{2}^{2}\right)}{{\left(\mu_{2}-\mu_{1}\right)}^{2}}$$

**Figure 3:**
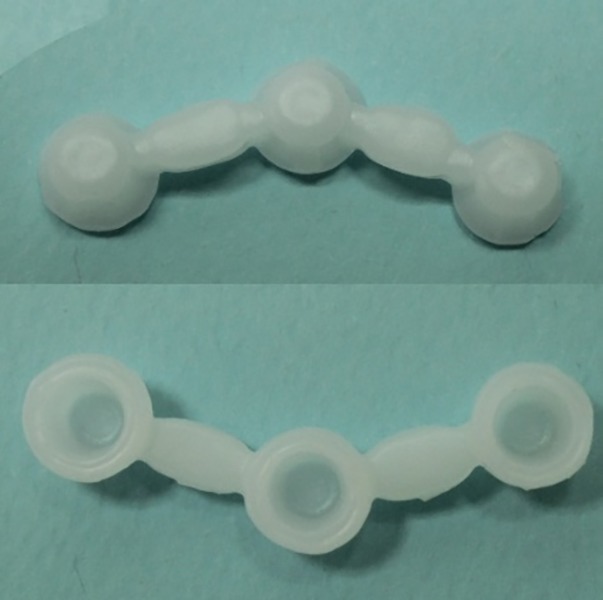
Wax pattern.

Thus, 20 samples were evaluated in group 1 and 20 samples in group 2. To standardize the frameworks fabricated by the conventional casting method and eliminate the errors related to impression making and pouring the casts, the original reference model was scanned with a light scanner (Ceramill Map400, Amann Girrbach, Germany) and the wax pattern was designed using the Ceramill Mind software (Ceramill Mind, Amann Girrbach, Germany). The copings had 1 mm of pattern thickness and 50 µm of cement space and were prepared up to 0.5 mm away from the margin. The three wax copings were connected to two pontics with 9-mm connectors to fabricate 5-unit bridges. Eventually, the patterns were fabricated using Ceramill Motion 2 milling machine (Ceramill Motion 2, Amann Girrbach, Germany) and wax blocks (Ceramill Wax, Amann Girrbach, Germany) (Figure 3).

Wax patterns were sprued using five sprues with 2.5-mm diameter and 7-mm length (Dentaurum Wax Wire on Roll, Dentaurum, Germany) and connected to the sprue bar (Dentaurum Sprue Bar, Dentaurum, Germany) and finally to the sprue former. Metal rings were then placed and a piece of cardboard with 1-mm thickness was used to compensate for metal shrinkage. The patterns were then coated with surfactant (Lubrofilm, Dentaurum, Germany) and dried with gentle air flow. The phosphate-bonded investment (Z4, Neirynck & Vogt, Belgium) was used for investing according to the manufacturer’s instructions and the patterns were cast (DUCATRON, Ugin Denraire, France) after wax burnout in an investment furnace (Sunny Therm, Koushafan Pars, Iran). The Ni-Cr alloy (New Cast, Yamahachi, Japan) consisting of 56% nickel, 20% chromium, 12% cobalt, 5% molybdenum, 2% beryllium, 1% titanium and 4% other elements was used in this study. The residual investment material was removed using a sandblaster (Basic mobil, Renfert, Germany) with 3-bar pressure and 125-µm aluminum oxide particles. The sprues were then cut using an abrasive disc and the frameworks were finished with abrasive stones (Red Mounted Point, Keystone Industries, USA). To fabricate frameworks for the second group, presintered soft Cr-Co blocks (Ceramill Sintron, Amann Girrbach, Germany) consisting of 66% cobalt, 28% chromium, 5% molybdenum, <1% silicium, <1% iron, <1% manganese and <1% carbon was used.

The designed bridges were used for the fabrication of wax patterns and non-sintered blocks were milled by the CAD/CAM machine accordingly.

The samples were sintered in a sintering furnace (Ceramill Argotherm 2, Amann Girrbach, Germany) at 1300°C using argon gas, and the attachments were separated after sintering using an abrasive disc.

Measurements were made using a bridge-type CMM (Zeiss WMM 850, Zeiss, Germany) in an isolated room in terms of temperature, humidity and dust. The accuracy of the CMM was 2.9+ L/300 µ (L=measurement length) in x and y axes and 3.5+ L/300 µ in z axis.

**Figure 4:**
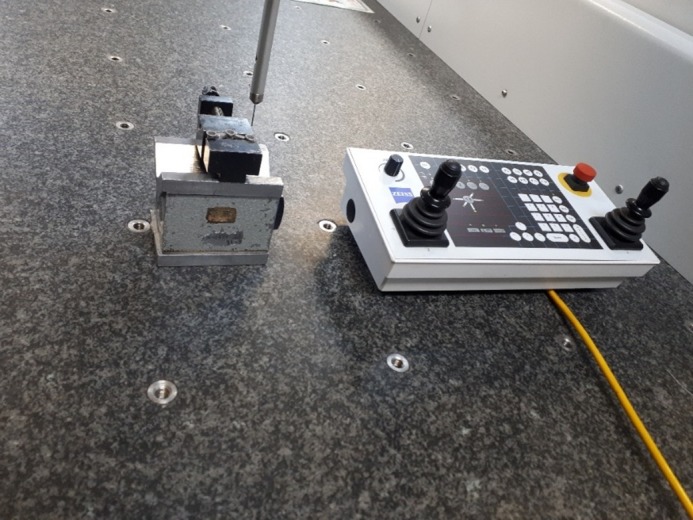
Coordinate measuring machine.

The smallest probe of CMM with 1-mm diameter was moved by the operator using the software (Zeiss Calypso, Zeiss, Germany) (Figure 4) and the position of each abutment and the respective retainers was recorded three-dimensionally (in x, y and z axes) by contacting different points in the sample.

**Figure 5:**
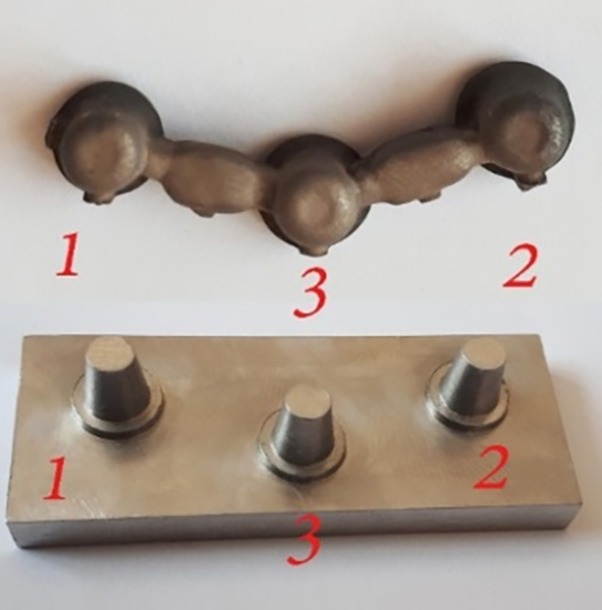
Coding of abutments and retainers.

The method used for measurements and assessment of the fit of frameworks has been previously used in some studies.^27,30-31^ First, the retainers and abutments were coded as shown in Figure 5 in order for the frameworks to have the same position in all the measurements (for the purpose of comparison).

Next, four points in the shoulder area (finish line) of each sample (both frameworks and the original model) were selected by a probe and the CMM defined a plane for each sample according to these four points (this plane was at the finish line of samples). This plane was then positioned parallel to the horizon in the software in order for the samples to be comparable. By doing so, the same plane was used for measurements in all the samples.

In the next step, the probe tip contacted the internal surface of framework retainers and external surface of abutments of the original model, and a central point (in the same defined plane) was determined for each of these abutments.

To assess the changes in frameworks compared to the original model, each framework had to be seated on the original model and share a point with it (in the same plane). This was done to compare the changes in position of other points of the framework relative to the original model (in other words, a similar seating position had to be defined for all the frameworks). For this purpose, the central points of the first (#1) abutment in the original model and #1 retainer in each framework were positioned at a point with x=0, y=0 and z=0 coordinates and this point was defined in the software for all the samples. At this time, the original model and all the frameworks had one shared plane (horizontal plane) and one shared point (central point of abutment #1). The dimensional changes of frameworks were then evaluated at this seating position. However, it should be noted that the CMM recorded the amount of deviation of these points in all the three dimensions before changing the position of central points of retainers and abutment #1 to the point 0, 0, 0, in order to allow comparison of retainers and abutment #1. Eventually, the changes in the position of central point of each retainer relative to the corresponding retainer in each of the three axes were measured and recorded. According to a previous study,^31^ the three-dimensional changes were determined using the following formula (2):

[2] $$3D=\sqrt{x^{2}+y^{2}+z^{2}}$$

It should be noted that two experienced operators made all the measurements and the mean values were recorded. Data were analyzed with descriptive statistics. The independent sample Student’s t-test or its non-parametric equivalent, one-way ANOVA and Tukey test were used to compare the two groups. Data were analyzed using SPSS 16 (SPSS Inc., IL, USA) at 5% level of significance.

## Results

Table 1 presents the changes in frameworks compared to the original model in each of the corresponding retainers (in the two groups) in mm.

**Table 1:** Mean dimensional changes of the three retainers in each framework using one-way ANOVA

**Variable**	**Retainer**	N*	**Mean**	**SD**	**Minimum**	**Maximum**	**F**	**P-value**
**Delta x**	1	40	0.02	0.06	0.00	0.30	71.68	<0.001
	2	40	0.22	0.11	0.00	0.63		
	3	40	0.07	0.05	0.01	0.25		
	Total	120	0.10	0.11	0.00	0.63		
**Delta y**	1	40	0.00	0.01	0.00	0.06	37.19	<0.001
	2	40	0.00	0.01	0.00	0.03		
	3	40	0.06	0.06	0.00	0.26		
	Total	120	0.02	0.04	0.00	0.26		
**Delta z**	1	40	0.08	0.09	0.00	0.50	5.69	<0.001
	2	40	0.37	0.47	0.00	2.23		
	3	40	0.26	0.46	0.00	2.31		
	Total	120	0.24	0.40	0.00	2.31		
**Delta 3D**	1	40	0.08	0.10	0.00	0.58	10.76	<0.001
	2	40	0.47	0.44	0.15	2.25		
	3	40	0.31	0.45	0.06	2.31		
	Total	120	0.29	0.40	0.00	2.31		

*There were 40 retainers in each group.

One-way ANOVA was applied to compare changes among the three retainers, which showed that dimensional changes of each retainer were significantly different from those of other retainers (P<0.001).

Tukey HSD test was used for pairwise comparisons of changes in framework retainers compared to the original model; the results of these comparisons are presented in Table 2. The results showed that:

**Table 2:** Pairwise comparison of changes in framework retainers compared to the original model using Tukey HSD test

**Variable**	**Changes in Retainer**	**Compared to retainer**	**Mean Difference**	**Std. Error**	**P-value**
**Delta x**	1	2	-0.20	0.02	<.001
		3	-0.05	0.02	<.001
	2	3	0.14	0.02	<.001
**Delta y**	1	2	0.00	0.01	1.00
		3	-0.06	0.01	<.001
	2	3	-0.06	0.01	<.001
**Delta z**	1	2	-0.29	0.09	<.001
		3	-0.18	0.09	0.09
	2	3	0.10	0.09	0.46
**Delta 3D**	1	2	-0.38	0.08	<.001
		3	-0.22	0.08	0.02
	2	3	0.16	0.08	0.13

1. All the three retainers had significant differences from each other in terms of dimensional changes in the x axis (P<0.001).
2. Regarding dimensional changes in the y axis, changes in retainer #1 were not significantly different from those in retainer #2 (P=1.00) but they were significantly different from those in retainer #3 (P<0.001). Moreover, changes in retainer #2 compared to those in retainer #3 were statistically significant (P<0.001).
3. In terms of dimensional changes in the z axis, changes in retainer #1 compared to those in retainer #2 were statistically significant (P<0.001), but no significant difference was noted between retainer #1 and retainer #3 in this respect (P=0.09). Changes in retainer #2 compared to retainer #3 were not significant (P=0.46).
4. In terms of three-dimensional changes, changes in retainer #1 were significantly different from those in retainers #2 and #3 (P<0.001). Moreover, changes in retainer #2 were not significant compared to those in retainer #3 (P=0.13).

Student’s t-test was used to compare changes in the retainers of the two groups; the results are summarized in Table 3. The results showed that the difference in dimensional changes of the corresponding retainers in the two groups was not statistically significant for any of the four measured variables (P>0.05).

**Table 3:** Comparison of the means and standard deviations of dimensional changes in the two groups using Student’s t-test (n=60)

**Variable**	**Group**	**Mean**	**SD**	**t**	**P-value**
**Delta x**	Conventional	0.10	0.11	-0.02	0.98
	Ceramill Sintron	0.10	0.12		
**Delta y**	Conventional	0.02	0.02	-1.56	0.12
	Ceramill Sintron	0.03	0.05		
**Delta z**	Conventional	0.22	0.33	-0.47	0.64
	Ceramill Sintron	0.25	0.46		
**Delta 3D**	Conventional	0.27	0.33	-0.49	0.62
	Ceramill Sintron	0.30	0.46		

Student’s t-test was used to compare longitudinal and transverse changes in the frameworks and the amount of marginal gap at the site of retainers #2 and #3; the results are summarized in Table 4. As shown, the two groups of frameworks were significantly different in terms of transverse changes (P<0.05) but longitudinal changes and changes in the amount of marginal gap at the three areas were not significantly different between the two groups (P>0.05).

**Table 4:** Comparison of longitudinal and transverse changes of frameworks and the amount of marginal gap measured at the site of retainers #2 and #3 using Student’s t-test (n=20)

	**Group**	**Mean**	**SD**	**t**	**Sig. (2-tailed)**
**Longitudinal changes**	Conventional	0.23	0.08	0.55	0.58
	Ceramill Sintron	0.21	0.14		
**Transverse changes of framework**	Conventional	0.04	0.02	-2.13	0.04
	Ceramill Sintron	0.08	0.07		
**Gap in retainer 1**	Conventional	0.08	0.05	0.35	0.62
	Ceramill Sintron	0.07	0.11		
**Gap in retainer 2**	Conventional	0.39	0.49	0.29	0.77
	Ceramill Sintron	0.34	0.47		
**Gap in retainer 3**	Conventional	0.18	0.21	-1.09	0.28
	Ceramill Sintron	0.34	0.62		

## Discussion

According to the current results, the null hypothesis of this study was refuted since all the three retainers in the two groups showed significant dimensional changes in all the three dimensions compared to the original model. In terms of dimensional changes in the three axes, the highest mean dimensional changes were noted in the vertical axis (z). Dimensional changes in the z axis are equal to the amount of gap at the framework margin and are more important clinically and with regard to long-term prognosis of treatment.^11-16^ Thus, dimensional changes in the z axis should always be minimal to prevent complications such as cement washout and subsequent periodontal problems, secondary caries and pulpitis.^14-19^

In our study, all the frameworks showed marginal gaps (dimensional changes in the z axis) in all the corresponding retainers and the highest mean amount of gap was noted in retainer #2 (the last retainer) while the lowest amount of gap was noted in retainer #1 (first retainer); this difference was statistically significant.

Gradual increase in marginal gap from the first to the last retainer indicates a vertical rotational pattern in frameworks, which is referred to as rocking in the clinical setting. This rotation might be eliminated following seating of framework and its adjustment in the clinical setting or may necessitate repeating of the fabrication of framework. Moreover, this finding might indicate that by elongation of framework, the vertical gap created in the posterior region is maximized, which increases the degree of rotation or rocking and vice versa.

Comparison of frameworks fabricated by the two methods revealed that the marginal gap (dimensional changes in z axis) was not significantly different in all the measured areas, and both groups had evident rocking. This finding is in agreement with the results of previous studies that compared the marginal fit and marginal gap of restorations fabricated by the conventional casting method and those fabricated by milling of Ceramill Sintron blocks.^10,32^ The results of previous investigations as well as the current study showed that the new method of restoration fabrication has no significant effect on marginal gap, and the marginal gap of restorations fabricated by this method is similar to that of conventionally fabricated restorations.

Assessment of dimensional changes of frameworks in the x axis, which indicates the longitudinal changes of frameworks, revealed that dimensional changes in all the three retainers and all the frameworks were statistically significant such that the maximum and minimum longitudinal changes were noted in retainers #2 (the farthest retainer) and #1 (the closest retainer), respectively.

These observations highlight the fact that by elongation of framework, longitudinal changes of framework increase and vice versa. Moreover, longitudinal changes of frameworks were not significantly different between the two groups; which means that the new method of framework fabrication has no advantage over the conventional method with regard to longitudinal changes of frameworks. This finding was in accordance with that of Kocaagaoglu et al in 2016;^33^ they reported no significant difference between the two groups regarding dimensional changes in the axial dimension.

Assessment of dimensional changes in the y axis, which indicate changes in framework arch, revealed the least dimensional changes in this axis (compared to other coordinates). Moreover, dimensional changes in all the three retainers and frameworks were statistically significant; in this context, the greatest transverse changes were noted in retainer #3 (retainers at the center of framework and out of the arch curve), which were significantly different from changes in retainers #1 and #2. However, transverse changes were the same in retainers #1 and #2 and were not significantly different. These observations indicate that in curved frameworks, transverse changes in the framework increase and vice versa. In other words, if the original model abutments are aligned in a straight line (in transverse dimension), the fabricated framework would have minimal (statistically insignificant) transverse changes.

Frameworks in the two groups had significant differences in terms of transverse changes, and this was an interesting finding of our study. The results revealed that the conventionally fabricated frameworks had mean lower transverse changes than milled frameworks using the Ceramill Sintron blocks. Therefore, the new method of fabrication of frameworks (the milling method using the Ceramill Sintron blocks) does not have any advantage with regard to minimizing the transverse changes of frameworks and even results in greater changes compared to the original model.

To the best of authors’ knowledge, no previous study has evaluated three-dimensional changes of milled frameworks using the Ceramill Sintron blocks, and the available studies have mostly evaluated single-unit restorations. Thus, comparison of our findings with those of previous studies was not feasible.

In general, none of the prostheses fabricated in this study had passive fit; this finding was in agreement with that of previous studies on the fit of dental prostheses or implants, highlighting the fact that despite recent advances in designing and manufacturing techniques of dental restorations, they are still incapable of achieving ideal (100%) adaptation and passive fit.^31,34-39^

## Conclusion

Within the limitations of this study, the following results were obtained:

1. All the three retainers in both groups exhibited significant dimensional differences from the original model in all the three dimensions.
2. The highest and the lowest dimensional changes were noted in the z (vertical) and y (transverse) axes, respectively.
3. By elongation of frameworks, dimensional changes in all the three axes (i.e. longitudinal and transverse changes and marginal gap) increase.
4. Comparison of the two study groups revealed no significant differences in longitudinal changes or marginal gaps.
5. The two groups exhibited a significant difference in transverse changes, and the conventionally fabricated frameworks showed significantly less transverse changes.
6. None of the prostheses fabricated in this study had ideal adaptation or passive fit.

## Acknowledgement

This article was written based on a dissertation for a specialty degree in prosthodontics. The authors would like to extend their gratitude to the Deputy of Research at Hamadan University of Medical Sciences and the Dental Research Center for the financial support provided.

## Authors’ contributions

All the authors have contributed to the concept and design of the study. AI, FV and AS supervised the procedural steps of the experiment. BFA contributed to the collection of data. The statistical analyses and interpretation of data were carried out by BFA and GR. AI and BFA drafted the manuscript. All the authors critically revised the manuscript for intellectual content. All the authors have read and approved the final manuscript.

## Funding

Financial support for this study was provided by Deputy of Research and the Dental Research Center, both at Hamadan University of Medical Sciences.

## Competing interests

The authors declare no competing interests with regards to the authorship and/or publication of this article.

## Ethics approval

Not applicable.

## References

[1] Wataha JC (2002). Alloys for prosthodontic restorations. J Prosthet Dent.

[2] Hildebrand HF, Veron C, Martin P (1989). Nickel, chromium, cobalt dental alloys and allergic reactions: an overview. Biomaterials.

[3] Wataha JC, Lockwood PE (1998). Release of elements from dental casting alloys into cell-culture medium over 10 months. Dent Mater.

[4] Lee DH, Lee BJ, Kim SH, Lee KB (2015). Shear bond strength of porcelain to a new millable alloy and a conventional castable alloy. J Prosthet Dent.

[5] Eliasson A, Arnelund CF, Johansson A (2007). A clinical evaluation of cobalt-chromium metal-ceramic fixed partial dentures and crowns: A three- to seven-year retrospective study. J Prosthet Dent.

[6] Anadioti E, Aquilino SA, Gratton DG, Holloway JA, Denry IL, Thomas GW (2015). Internal fit of pressed and computer-aided design/computer-aided manufacturing ceramic crowns made from digital and conventional impressions. J Prosthet Dent.

[7] Bidra AS, Taylor TD, Agar JR (2013). Computer-aided technology for fabricating complete dentures: systematic review of historical background, current status, and future perspectives. J Prosthet Dent.

[8] Yoon TH, Madden JC, Chang WG (2013). A technique to restore worn denture teeth on a partial removable dental prosthesis by using ceramic onlays with CAD/CAM technology. J Prosthet Dent.

[9] Kim KB, Kim JH, Kim WC, Kim HY, Kim JH (2013). Evaluation of the marginal and internal gap of metal-ceramic crown fabricated with a selective laser sintering technology: two- and three-dimensional replica techniques. J Adv Prosthodont.

[10] Park JK, Kim HY, Kim WC, Kim JH (2016). Evaluation of the fit of metal ceramic restorations fabricated with a pre-sintered soft alloy. J Prosthet Dent.

[11] Holmes JR, Bayne SC, Holland GA, Sulik WD (1989). Considerations in measurement of marginal fit. J Prosthet Dent.

[12] Jahangiri L, Wahlers C, Hittelman E, Matheson P (2005). Assessment of sensitivity and specificity of clinical evaluation of cast restoration marginal accuracy compared to stereomicroscopy. J Prosthet Dent.

[13] Limkangwalmongkol P, Chiche GJ, Blatz MB (2007). Precision of fit of two margin designs for metal-ceramic crowns. J Prosthet.

[14] Quante K, Ludwig K, Kern M (2008). Marginal and internal fit of metal-ceramic crowns fabricated with a new laser melting technology. Dent Mater.

[15] Stephen F. Rosenstiel MFL, Junhei Fujimoto. Contemporary Fixed Prosthodontics. 5th ed. China: ELSEVIER; 2016.

[16] Vigolo P, Fonzi F (2008). An in vitro evaluation of fit of zirconium-oxide-based ceramic four-unit fixed partial dentures, generated with three different CAD/CAM systems, before and after porcelain firing cycles and after glaze cycles. J Prosthet.

[17] Kokubo Y, Ohkubo C, Tsumita M, Miyashita A, Vult von Steyern P, Fukushima S (2005). Clinical marginal and internal gaps of Procera AllCeram crowns. J Oral Rehabil.

[18] Mitchell CA, Pintado MR, Douglas WH (2001). Nondestructive, in vitro quantification of crown margins. J Prosthet Dent.

[19] Soriani NC, Leal MB, Paulino SM, Pagnano VO, Bezzon OL (2007). Effect of the use of die spacer on the marginal fit of copings cast in NiCr, NiCrBe and commercially pure titanium. Braz Dent J.

[20] Sabouhi M, Hedayatipanah M. The Comparison of Vertical Margin Discrepancy in Casting Fabricated with Metal Ring, Ringless and Metal Ring with Hygroscopic Expansion Investment Systems. Avicenna J Dent Res 2015; 7(2): e24834. http://ajdr.umsha.ac.ir/Abstract/ajdr-112.

[21] Boeddinghaus M, Breloer ES, Rehmann P, Wostmann B (2015). Ac-curacy of single-tooth restorations based on intraoral digital and conventional impressions in patients. Clin Oral Investig.

[22] Molin M, Karlsson S (1993). The fit of gold inlays and three ceramic inlay systems A clinical and in vitro study. Acta Odontol Scand.

[23] Sorensen JA (1990). A standardized method for determination of crown margin fidelity. J Prosthet Dent.

[24] Kim EH, Lee DH, Kwon SM, Kwon TY (2016). A microcomputed tomography evaluation of the marginal fit of cobalt-chromium alloy copings fabricated by new manufacturing techniques and alloy systems. J Prosthet Dent.

[25] Kim KB, Kim JH, Kim WC, Kim JH (2014). Three-dimensional evaluation of gaps associated with fixed dental prostheses fabricated with new technologies. J Prosthet Dent.

[26] Vojdani M, Torabi K, Atashkar B, Heidari H, Torabi Ardakani M (2016). A Comparison of the Marginal and Internal Fit of Cobalt- Chromium Copings Fabricated by Two Different CAD/CAM Systems (CAD/ Milling, CAD/ Ceramill Sintron). J Dent.

[27] Jemt T, Rubenstein JE, Carlsson L, Lang BR (1996). Measuring fit at the implant prosthodontic interface. J Prosthet Dent.

[28] Paniz G, Stellini E, Meneghello R, Cerardi A, Gobbato EA, Bressan E (2013). The precision of fit of cast and milled full-arch implant-supported restorations. Int J Oral Maxillofac Implants.

[29] Ma L, Xu T, Lin J (2009). Validation of a three-dimensional facial scanning system based on structured light techniques. Comput Methods Programs Biomed.

[30] Hjalmarsson L, Ortorp A, Smedberg JI, Jemt T (2010). Precision of fit to implants: a comparison of Cresco and Procera(R) implant bridge frameworks. Clin Implant Dent Relat Res.

[31] Eliasson A, Wennerberg A, Johansson A, Ortorp A, Jemt T (2010). The precision of fit of milled titanium implant frameworks (I-Bridge) in the edentulous jaw. Clin Implant Dent Relat Res.

[32] Presotto AG, Bhering CL, Mesquita MF, Barao VA (2017). Marginal fit and photoelastic stress analysis of CAD-CAM and overcast 3-unit implant-supported frameworks. J Prosthet Dent.

[33] Kocaagaoglu H, Kilinc HI, Albayrak H, Kara M (2016). In vitro evaluation of marginal, axial, and occlusal discrepancies in metal ceramic restorations produced with new technologies. J Prosthet Dent.

[34] Jemt T, Back T, Petersson A (1999). Precision of CNC-milled titanium frameworks for implant treatment in the edentulous jaw. Int J Prosthodont.

[35] Riedy SJ, Lang BR, Lang BE (1997). Fit of implant frameworks fabricated by different techniques. J Prosthet Dent.

[36] Ortorp A, Jemt T, Back T, Jalevik T (2003). Comparisons of precision of fit between cast and CNC-milled titanium implant frameworks for the edentulous mandible. Int J Prosthodont.

[37] Takahashi T, Gunne J (2003). Fit of implant frameworks: an in vitro comparison between two fabrication techniques. J Prosthet Dent.

[38] Al-Fadda SA, Zarb GA, Finer Y (2007). A comparison of the accuracy of fit of 2 methods for fabricating implant-prosthodontic frameworks. Int J Prosthodont.

[39] Cheshire PD, Hobkirk JA (1996). An in vivo quantitative analysis of the fit of Nobel Biocare implant superstructures. J Oral Rehabil.

